# Editorial: Qualitative Methods for Studying Groups

**DOI:** 10.3389/fpsyg.2022.946575

**Published:** 2022-06-15

**Authors:** Francesca Alby, Francesco Arcidiacono, Maria Fernandes-Jesus, Terri Mannarini, Laura Lucia Parolin, Liisa Voutilainen

**Affiliations:** ^1^Department of Social and Developmental Psychology, Sapienza University of Rome, Rome, Italy; ^2^Research Department, Haute École Pédagogique BEJUNE, Biel, Switzerland; ^3^School of Education, Language and Psychology, York St John University, York, United Kingdom; ^4^Department of Human and Social Sciences, University of Salento, Lecce, Italy; ^5^Department of Language and Communication, University of Southern Denmark, Slagelse, Denmark; ^6^Faculty of Social Sciences, University of Helsinki, Helsinki, Finland

**Keywords:** groups, qualitative methods, constructivist epistemology, emic point of view, situatedness, action-research

Research on small groups in social and behavioral sciences involves many studies and considerable theoretical insight (Levine and Moreland, 1990; McGrath et al., 2000). Interest in exploring social processes within small groups has a long history, yet in some areas—namely in social psychology—it has decreased over time, due to a shift to the study of intergroup relations (Wittenbaum and Moreland, 2008). Traditionally, this bulk of research has been characterized by quantitative methods, even though in the last two decades qualitative research has progressively entered this field (Emich et al., 2020), and the need for qualitative analyses of micro aspects of group processes has been acknowledged as an opportunity to be seized (Keyton, 2016). However, the most part of small group research has been driven, and continues to be driven by a positivistic epistemology and with an experimental approach to data collection that does not pay attention to emic views and to groups' relations to contexts. A different approach has been developed within qualitative, especially ethnographic, research. A large tradition of microanalytic studies has focused on discursive and multimodal interactions within groups in a variety of contexts, highlighting, among other things, the mediating role of artifacts and processes of embodiment in collective action and interaction (see, among many others, Goodwin, 1994; Luff et al., 2000; Streeck et al., 2011). Within a practice-based approach, several authors explored groups as the result of a nexus of practices, highlighting them not as social objects defined by rules, roles, and mental contents, as in the traditional rationalist view, but as forms of joint practicing that collectively shape social and psychological processes such as knowing, meaning-making, identity-forming, and other order-producing activities (Chaiklin and Lave, 1993; Schatzki, 2001; Gherardi, 2009; Nicolini, 2012).

Due to its emphasis on process instead of structure, this qualitative research literature rarely refers to the concept of group (see, however, Lave and Wenger, 1991; Hutchins, 1993; Wenger, 1998). Indeed, there is some disagreement also among group scholars about what is a “group” (Hollinghshead and Poole, 2012), hence we endorse an inclusive view of groups, since we believe that the concept of group can contribute to qualitative research by connecting micro and macro analyses, processes and structure, individual and society.

The idea of this Research Topic arises from this conviction and from this gap in the literature. By embracing a constructivist epistemology, and adopting a qualitative methodology, this Research Topic collection aims to capture the complexity of the phenomena that occur in groups in ordinary and institutional settings and to provide a detailed, situated understanding of the actors' experiences.

The articles published in the Research Topic build on our original vision, introducing diverse perspectives and group conceptions, documenting group activities in quite different contexts, including work, educational, healthcare, clinical, and community-based settings. The contributions explore a broad range of empirical phenomena.

Alby et al. (2022) analyze the narrative construction of psychoanalytic trainees' professional identity. Through thematic analysis of interviews, the study shows how professional identities are constructed as the outcome of learning practices and multiple group participations over time.

Annese et al. show the situational nature of an effective tutorship style in Whatsapp learning groups, based on an integrated quali-quantitative approach that combines thematic analysis and social network analysis.

Brito Rivera et al. show how group membership and social identity markers are discursively used by researchers to support emerging forms of collaboration and the creation of a common ground during a research-intervention in a hospital.

Bruzzone and Crevani describe a research-intervention process in which researchers, practitioners and older people were involved in co-creating a working method for supporting the introduction of welfare technologies for older people.

Kurilla's article provides a wide and comprehensive critical review of theories and perspectives for qualitative group research, and in particular conceptual devices for research on group communication.

Nitzan and Orkibi examine the experience of participation in arts-based groups for people with mental health conditions in a community rehabilitation program

Parolin and Pellegrinelli present an analysis of an aid practice addressed to the vulnerable population during the first peak of the pandemic. By showing how technologies have the potential to shape courses of action, their analysis—inspired by Actor-Network Theory—provides evidence of how groups, practices and sociomaterial networks are entangled.

Parrello et al. use a longitudinal and process-based qualitative approach to test the efficacy of the Multi-Vision Group - a modified version of the Balint Group - to support team reflexivity and members wellbeing within a non-profit organization operating to reduce socio-educational disparities.

Rania et al. explore reflective practices and group dynamics within online training activities with university students. The data collected through individual reflective practices and the transcripts of the group reflections are analyzed using grounded theory.

Saglietti and Marino focus on an intensive group-care context, i.e., group home for children, and examine the discursive accomplishment of intergroup relations. Based on ethnographic interviews, the authors discuss the co-construction of intergroup relations and ingroup bias.

Scaratti et al. rely on the concept of *liminality* to explore a particular formative group context in an extra-hospital Rehabilitation Center. The authors analyze conversational data from training sessions to illustrate how this particular context represents a liminal space in which a professional hybridization phenomenon occurs in connection to organizational restructuration, work uncertainty and professional challenges.

Wakke and Heller examine interactions in which students help each other with their learning during classroom instruction, forming groups in the process. The authors reveal how the problem definition is a key moment in the sequential and bodily-spatial unfolding of the help interaction.

In the paper of Jensen et al. an analytical framework for the analysis of organizational cognition is proposed to borrow from recent developments within distributed/ecological cognition.

Alhazmi and Kaufmann present the application of phenomenological qualitative methods to the analysis of cross-cultural experience in novel educational social contexts.

Through an analysis of a focus group discussion on TV series, Weiser-Zurmühlen proposes a methodological framework that combines conversation analysis, positioning theory and stance analysis in the study of group interactions. She argues that by considering both micro and macro contextual features, it is possible to gain a deeper understanding of interactional phenomena in groups.

The studies in this collection have some characteristics which are outlined in what follows.

## Situatedness of the Phenomena Under Scrutiny

Phenomena under scrutiny such as professional identity, tutorship style, group formation processes, organizational change, and others are considered in their relation to contexts and to the emic perspectives of the actors therein. Research settings include hospitals, community rehabilitation centers, group care homes, schools, and different kind of educational settings and workplaces. Social actors include doctors, nurses, managers, researchers, psychologists, educators, psychoanalytic trainees, university, elementary and high school students, technicians, developers, people with mental health conditions, “street teachers”, elderly people. Since in qualitative research data are not “found” but constructed within a researchers-participants relationship and through research instruments, these studies try to make them accessible as much as possible and accounted for in the analysis. By being faithful to research settings and instruments, and to actors' inside views, the studies in this collection aim to account for the situatedness of the phenomena that they explore.

## Qualitative Data Corpora

The studies in this collection rely on various types of qualitative data, which have the common characteristic of being discursive data, either interactional or textual. Among them we find naturally-occurring multi-party interactions (such as the ones collected in meetings or classrooms), focus group discussions, interviews, WhatsApp chats, observational reports or narrative reports produced by research participants.

## Theory Driven Methods

Almost all the studies collected rely on explicitly theory-driven methods, both as for the tools used to gather the data and as for the analytical procedures: phenomenology, ethnomethodology, activity theory, situated action theory, positioning theory, Actor-Network Theory, distributed cognition or a combination of them characterize this assortment.

## Multiple Analysis Procedures

Some research in the collection employs quantitative analyses (e.g., frequency analysis, social network analysis) of data gathered with qualitative methods such as ethnographic interviews or video recordings of meetings or WhatsApp chats. Other research makes use of qualitative analyses such as thematic analysis, discourse analysis, conversation analysis and multimodal analysis of interaction. Some authors rely on paper-and-pencil procedures while others on qualitative data analysis software (such as Nvivo).

## Multiple Perspectives on Groups

Groups are considered as quite different “objects” by the authors of the Research Topic. This variety is in line with the heterogeneity of definitions that more generally characterizes group research in social sciences.

In particular, groups are here understood as:

- devices for intervention, as in the case of groups set up for training, reflexive or rehabilitation/care purposes;
- forms of joint practice in contexts of private, educational and working life;
- technologically mediated collections, in which the interaction is distributed between various actors, in space and time and mediated by technologies and sociomaterial processes;
- discursive units, i.e., linguistic markers of collective social identities and group memberships used in communicative exchanges for local rhetorical and pragmatic purposes.

## Intertwining of Research and Action

Several articles describe cases of action-research, in which researchers and participants work together, within group settings, in activities aimed at facilitating reflection and change processes.

These studies' characteristics may or may not capture widespread trends in qualitative research on groups. However, this Research Topic certainly shows the existence of a fruitful field of research that crosses different disciplines and settings. While this Topic shows that attention is growing, the scope for future studies remains large, with many important understudied matters and their methodological implications.

We thank all the authors who contributed to this Research Topic with their findings, together with the reviewers and external editors whose help was essential in getting this collection successfully published.

## Author Contributions

All authors listed have made a substantial, direct, and intellectual contribution to the work and approved it for publication.

## Conflict of Interest

The authors declare that the research was conducted in the absence of any commercial or financial relationships that could be construed as a potential conflict of interest.

## Publisher's Note

All claims expressed in this article are solely those of the authors and do not necessarily represent those of their affiliated organizations, or those of the publisher, the editors and the reviewers. Any product that may be evaluated in this article, or claim that may be made by its manufacturer, is not guaranteed or endorsed by the publisher.
